# The Potential Therapeutic Effects of Low-Dose Ionizing Radiation in Alzheimer's Disease

**DOI:** 10.7759/cureus.23461

**Published:** 2022-03-24

**Authors:** Joubin Jebelli, Michael C Hamper, Danielle Van Quelef, Davian Caraballo, James Hartmann, James Kumi-Diaka

**Affiliations:** 1 Cancer Biology & Biochemistry, Charles E. Schmidt College of Medicine, Boca Raton, USA; 2 Neuroimmunology, Charles E. Schmidt College of Medicine, Boca Raton, USA; 3 Neurology, Florida Atlantic University, Boca Raton, USA; 4 Neurology, Charles E. Schmidt College of Medicine, Boca Raton, USA; 5 Biology & Biochemistry, Florida Atlantic University, Boca Raton, USA; 6 Biology & Biochemistry, Florida Atlantic University, Davie, USA

**Keywords:** therapeutics, radiation, novel treatment, neurodegeneration, alzheimer’s disease

## Abstract

Dementia is an umbrella term used to describe a loss of cognitive function which results in the interference of an individual's daily life and activities. The most common form of dementia is Alzheimer's disease. Alzheimer’s is classified as a progressive, debilitating neurodegenerative disease that results in disturbances to a patient’s higher executive function, memory, language, and visuospatial orientation. Despite extensive research on Alzheimer’s dementia, including both available and potential therapeutic modalities, this neurodegenerative disease is incurable and will continue to pose a major public health concern. Current treatment options for Alzheimer’s focus on symptom management and/or delaying the progression of the disease. Therefore, new treatment strategies must be developed to combat such a deadly disease. One field of medicine that has garnered significant interest from researchers to potentially treat Alzheimer’s is low-dose ionizing radiation. Various reports suggest that the brain’s exposure to low doses of ionizing radiation may serve as a therapeutic modality for combating neurodegenerative diseases, including Alzheimer’s dementia. This article serves as a review of the current available treatments for Alzheimer’s disease and discusses recent studies that provide evidence for the potential use of low-dose ionizing radiation as a therapeutic in the treatment of Alzheimer’s disease.

## Introduction and background

Dementia is an umbrella term used to describe a clinical diagnosis characterized by cognitive decline involving memory and at least one of the other cognitive domains, including social and visuospatial skills, executive functioning, complex attention, language, abstract thinking, personality, and praxis. Because of these cognitive deficits, patients diagnosed with dementia have difficulty with daily functions and are often dependent on others for daily activities [1]. The incidence of dementia increases with age and is becoming increasingly more common, both in the United States and the world, due to the growing aging population. Because dementia is currently incurable, the incidence rates will continue to increase, with an expected 131 million people worldwide being diagnosed with dementia by the year 2050 [2].

Dementia comes in many forms and is caused by damages and/or changes to the brain. The most common form of dementia is Alzheimer's disease, which will be the primary focus of this review article. Other common forms of dementia include vascular dementia, frontotemporal dementia, dementia of Lewy bodies, and Parkinson's disease dementia. There are multiple other diseases that can result in an increased risk of developing dementia, albeit less common, which include: Creutzfeldt-Jakob disease, Huntington disease, corticobasal degeneration, progressive supranuclear palsy, Leukoencephalopathies, and multiple-system atrophy [1]. This review will introduce Alzheimer's Disease, including a brief overview of its epidemiology, pathophysiology, and clinical characteristics before discussing current treatments regimens and a potential alternative therapeutic modality in low-dose ionizing radiation. 

## Review

Alzheimer's disease - an overview 

Incidence and Mortality

Alzheimer's disease (AD) is the most common form of dementia and accounts for at least 60-80% of dementia cases. As of 2021, an estimated 6.2 million Americans, ages 65 and older, are living with this disease. Barring the development of any novel therapeutics to prevent, slow, or cure Alzheimer's, this number will continue to increase with an approximated 13.8 million Americans being diagnosed with AD by 2060 [3].

Alzheimer's dementia is an incurable disease that continues to remain a major public health concern. Just in the year 2019, 121,499 Americans died from this disease, making AD the sixth leading cause of death in the United States [3]. Interestingly, recent studies suggest that AD may rank third, just behind heart disease and cancer, as the cause of death for elderly Americans [4]. AD is invariably progressive. The prognosis continues to remain dismal, with an average life expectancy of four to eight years for people ages 65 years and older. Some individuals with AD may live up to an additional 20 years after their initial diagnosis [5,6].

Pathophysiology of AD

The reported histopathological characteristics and the hallmarks of AD are the presence of extracellular aggregates (plaques) of insoluble β-amyloid peptide (Aβ) and intracellular aggregations of neurofibrillary tangles (NFTs), composed of hyperphosphorylated microtubule-associated τ (tau) proteins, in neuronal cytoplasm [7]. Studies have shown that Aβ plaques initially develop in the basal, temporal, and orbitofrontal neocortex regions of the brain. At later stages of the disease, these plaques spread/progress throughout the neocortex, hippocampus, amygdala, diencephalon, and basal ganglia. In very critical/serious cases of AD, Aβ is also found dispersed throughout the mesencephalon, lower brain stem, and cerebellar cortex [8]. Interestingly, it is believed that Aβ deposition triggers abnormal phosphorylation of τ, which leads to NFT formation and neurodegeneration [9]. NFTs, on the other hand, are often found in the locus coeruleus and both the transentorhinal and entorhinal regions of the brain. In the more critical stages of the disease, NFTs tend to spread to the hippocampus and neocortex [8]. 

There are still ongoing debates about the mechanisms by which the presence of Aβ and NFTs result in the cognitive decline of AD patients. Indeed, it is believed that the presence of these deposits result in excitotoxicity processes (a pathological process by which neurons are damaged and killed as a result of hyperactivity of excitatory neurotransmitters - mainly glutamate - in neuronal membranes), inflammation and depletion of energy and neuronal factors, and the collapse in calcium homeostasis. Consequently, this results in the atrophy and death of neurons involved in memory processing, language, learning, motor function, and other pertinent cognitive functions - thus leading to the clinical manifestations exhibited by AD patients [8].

Clinical Characteristics of AD

AD is classified as a progressive neurodegenerative disease with insidious onset [5]. The clinical manifestations of patients with AD include disturbances in memory, language, higher executive functions, and visuospatial orientation. Additionally, these patients often exhibit personality changes/mood disturbances and are prone to wandering, psychotic behavior (i.e., hallucinations, delusions, aggression, etc.), agitation, and sleep disturbances/abnormalities [10].

Individuals with Alzheimer's dementia typically experience subtle memory loss as well as a host of other neurological and psychological symptoms that change over a period of years. These symptoms often reflect the degree of damage to nerve cells in different parts of brain tissue. The pace at which symptoms of AD progress from mild to moderate and eventually to severe differs from individual to individual [11].

Patients who are in the mild stages of Alzheimer's dementia are often able to function independently. They are still likely to require some assistance with tasks to ensure their safety and to maximize their independence. Once in the moderate stages of Alzheimer's, which happens to be the longest stage of the disease, patients exhibit difficulty with communication and performing routine tasks. More likely than not, they will display personality and behavioral changes (i.e., depression, agitation, suspiciousness, and combative behavior), and their memory will progressively worsen. Patients may even have seizures and experience bladder and bowel incontinence. In the severe stages of Alzheimer's, individuals need assistance with routine daily activities and require full-time institutional care. At this stage, the effects of the disease are especially apparent and have put a heavy toll on the individual's physical health. They are malnourished, incapacitated, and bed-bound, making them vulnerable to sepsis, skin infections, and blood clots. These patients also have difficulty with swallowing, eating, and drinking. Death usually results in this stage and is often caused by malnutrition and aspiration pneumonia. The typical clinical duration of AD is normally between eight to 10 years, but this is also contingent on the severity of the disease and the age of the patient. However, the symptoms exhibited by the patient can range anywhere between one to 25 years [11].

Current treatment modalities for AD

Despite extensive research in the treatment and management of AD, it is highly unlikely that any one drug or intervention will successfully treat this disease. Current approaches focus on managing symptoms and/or merely delaying disease progression [12]. Patients diagnosed with AD will often encounter a multifactored tailored therapeutic regimen consisting of caregiver support, behavioral approaches (i.e., exercise and music therapy, cognitive therapy, established routines, communicative strategies, etc.), an open line of communication with physicians and caretakers, and pharmacological interventions [13]. The remainder of this section will discuss the pharmacological interventions for AD.

Currently, there are four FDA-approved medications for the treatment of AD. Three of these medications - donepezil, rivastigmine, and galantamine - are acetylcholinesterase (AChE) inhibitors. The fourth drug is memantine, an n-methyl-d-aspartate (NMDA) receptor antagonist. Interestingly, tacrine, which is another AChE inhibitor, became the first FDA-approved drug for treating AD. However, this medication was discontinued in 2013 due to an association with hepatotoxicity and a multitude of other side-effects including nausea, vomiting, diarrhea, anxiety, and insomnia [14].

The AChE inhibitors are known as the "first-line" medications for the treatment of AD [15]. Because decreased cholinergic function is linked to cognitive impairments in patients with AD, donepezil, rivastigmine, and galantamine are often utilized to restore the cholinergic pathway. These AChE inhibitors act by binding to and inhibiting acetylcholinesterase and, to a lesser extent, butyrylcholinesterase, thus increasing the levels of acetylcholine (a neurotransmitter that plays a critical role in memory and other cognitive functions) at the synapse [14]. In this regard, the AChE inhibitors - donepezil and galantamine - inhibit selectively and reversibly to acetylcholinesterase. Rivastigmine, on the other hand, is a "pseudo-irreversible" inhibitor of both acetylcholinesterase and butyrylcholinesterase [13].

All three of the AChE inhibitors have proven to be therapeutic for AD patients such that they delay cognitive decline and stabilize or even improve cognition. Clinical studies have shown there are no significant differences in the efficacy of these three medications. It is, however, recommended for patients with AD to be placed on these AChE inhibitors as soon as possible, preferably right after their initial diagnosis. In fact, it is well documented that patients, who were placed on AChE inhibitors six months after their initial diagnosis, exhibited a more rapid cognitive decline than those who started taking the medications immediately. The FDA has now approved donepezil and rivastigmine for the treatment of mild, moderate, and severe AD. Galantamine has been approved for the treatment of mild and moderate AD [13].

Memantine is a noncompetitive low-affinity NMDA-receptor antagonist that affects glutamatergic transmission. This drug acts by blocking the effects of excessive glutamate stimulation at the NMDA receptors, thus preventing an excess of downstream calcium influx and oxidative stress. This results in the prevention of excessive excitotoxicity and neuronal cell death, which are reported to contribute to the pathogenesis of AD [14]. Interestingly, glutamate is typically elevated in AD and is believed to be a consequence of inefficient removal mechanisms at the synaptic cleft. This abnormal buildup of glutamate leads to the overactivation of NMDA receptors, resulting in chronic excitotoxicity [14,16]. Memantine has been approved for the treatment of moderate to severe AD, either as a monotherapy or in combination with the AChE inhibitors. Patients receiving this medication tend to display both short- and long-term benefits, such that it alleviates behavioral/psychological symptoms and improves their cognition and daily living [13].

Combination therapy, involving the administration of memantine with the AChE inhibitors, has also been tested and now provides another viable treatment option. In fact, a fixed combination of memantine and donepezil, in capsule form, was first approved in 2014 for the treatment of moderate to severe AD. Current scientific evidence suggests but does not definitely prove, that the co-administration of memantine with an AChE inhibitor provides for an even more effective treatment regimen. In fact, a recent meta-analysis conducted by the European Academy of Neurology illustrated additional evidence for the effectiveness of combination therapy. In comparison to monotherapy, administering the combination therapy of drugs yielded better results in the patient's behavior, mood, and cognitive function [17]. Similar results were observed in another meta-analysis of seven trials in patients with mild to severe AD [18]. It is now believed that co-administering memantine with an AChE inhibitor can be more beneficial as they have complementary mechanisms of action (additive effects) without any increase in adverse side-effects [13,19].

Current Landscape for AD Treatment

Currently, many of the new therapies being tested lack efficacy and/or possess unacceptable side effects. Moreover, many of the novel therapies being tested in animal models often lack predictive value in humans [12]. The medications currently in the market for Alzheimer's treatment have been demonstrated to only slow down disease progression and may possibly delay the onset of symptoms. These drugs are not curative by any means and have modest therapeutic effects. Many researchers, in fact, still question the effectiveness of these drugs to this day [12].

New treatment strategies must be established to combat such a deadly disease. Having an increased understanding of the pathophysiology of AD could possibly assist in accomplishing such a difficult feat. Of course, no one therapy will probably ever cure this disease. Utilizing combination therapies will likely be the preferred course of action and humanity's best bet to manage AD. There is one field in medicine; however, that is garnering a lot of attention from researchers and may very well be the next established therapeutic modality for AD. This field involves low doses of ionizing radiation (LDIR) and will be the remaining focus of this review.

Low-dose ionizing radiation 

What is Low-Dose Ionizing Radiation?

In accordance with the Biological Effects of Ionizing Radiation (BEIR) VII report, which is the seventh journal in a series of publications from the National Academies concerning radiation health effects, LDIR is classified as radiation dosages that range from values near 0 up to about 100 mSV (100 mGy). Humans are regularly exposed to LDIR, including an average annual background radiation of 3 mGy. Additionally, people are exposed to radioactive materials that are widely used in industrial applications and to that of diagnostic equipment, such as CT scans, in which the radiation dose ranges between 3 to 20 mGy [20]. 

Since the discovery of ionizing radiation, the linear-no-threshold (LNT) hypothesis was established as a guideline for radiation protection standards. However, this model proposed that even a very small dose of ionizing radiation can be harmful to living organisms and initiate carcinogenesis or increase the risk of other diseases [21,22]. Despite the near-universal adoption of the LNT model, it remains highly controversial among scientists and healthcare professionals. As many researchers continue to point out, the LNT model essentially neglects the fact that life developed and evolved on Earth in conditions of natural background radiation levels. With the rise of aerobic organisms, these specimens developed evolutionary adaptive responses to survive such living conditions, including possessing DNA repair mechanisms and maintaining cellular responses to eliminate damaged cells. They also possessed powerful defenses against metabolically induced reactive oxygen species (ROS), which helped contribute to their overall survival [20,21].

Over the past several decades, extensive research has been performed on the effects of radiation in a wide variety of species. These studies provided substantial scientific evidence that the biological responses to LDIR are vastly distinct from that of high-dose radiation exposure. Indeed, it was the results of these experiments that led to the development of radiation hormesis, a theory that emphasizes the ability of biological systems to respond positively to exposures of LDIR, such that there is a stimulation in the activation of repair mechanisms [23]. Interestingly, numerous studies utilizing both animal and human models revealed that LDIR is able to stimulate the proliferation of normal cells, promote enzymatic repair and the reparation of normal tissues, enhance the immune response, suppress the aging process, and even prevent or delay carcinogenesis/cancer progression [24-26]. LDIR also activates DNA repair mechanisms and acts upon cells that participate in the inflammatory response, thus producing anti-inflammatory effects [27]. Moreover, LDIR has been shown to stimulate each component of the protective systems of antioxidant prevention, thereby diminishing genomic instability and averting free radicals and/or reactive oxygen species induced damage to normal cells [28].

*Effect of LDIR on the Central Nervous System*

While radiation does provide for one of the most important diagnostic tools and therapeutic modalities in modern medicine, there is still major contention surrounding the effects of radiation on the central nervous system (CNS). A recent meta-analysis investigating the exposure of brain tissue to high-doses of ionizing radiation revealed the expression and release of biochemical mediators of neuroinflammation, such as ROS and proinflammatory cytokines, resulting in the destruction of tissues [29]. Exposure to such doses of radiation also affected neurovascular permeability and damaged nucleic acids [25,30-32,33]. Unfortunately, high-dose cranial radiation therapy can cause profound morphological and functional changes in brain tissues. Indeed, it is because of such changes that patients, undergoing high-dose radiation therapy, will often encounter various side effects including declining cognitive function, memory and attention deficits, speech problems, fatigue/weakness, paresthesia, headaches, and seizures. Additionally, many of these patients encounter acute side-effects (i.e., skin burns and acute radiation syndrome), as well as long-term health problems, most notably intracranial tumors and cardiovascular diseases [34,35]. 

Although the effects of high-dose cranial radiation therapy have been thoroughly investigated, the neurobiological effects and mechanisms of the brain's response to LDIR remain somewhat in question. In fact, there are significantly fewer published studies, as compared to high-dose radiation therapy, investigating the effects of LDIR on the CNS [36]. However, a predominant theme in much of the scientific literature pertaining to LDIR exposure and the brain illustrates that low dose irradiation may be protective in nature and even beneficial [30]. A particular research group, who provided some of the earliest insights into the acute effects of low dose radiation on the brain, showed that LDIR activates protective mechanisms in rat brains by reducing lipid peroxides through the induction of antioxidant activity [37]. Using mouse models, it was demonstrated that brain exposure to LDIR can stimulate neural stem cell proliferation, thus promoting neurogenesis in the hippocampus and ultimately improving learning [38]. Another study involving mouse models with Parkinson's disease (PD) showed that when mice were exposed to LDIR, it resulted in reduced oxidative stress, mitochondrial dysfunction, and the induction of apoptosis in affected neuronal cells. The results suggested that LDIR could play an important role in attenuating the effects of PD [39].

There is now a growing body of evidence that suggests CNS exposure to LDIR produces responses consistent with radiation hormesis. Albeit these experiments utilized animal models, the results were promising and illustrated that LDIR may not induce deleterious alterations in cognition, cell functioning, DNA, and gene expression [40]. Moreover, these studies suggest that LDIR can actually induce repair mechanisms against CNS pathology, stimulate defenses against neuroinflammation, and mitigate oxidative stress [30]. Additionally, LDIR confers protection to cell functioning, synapses, molecular structures, and key mechanisms like neurogenesis [26,41]. Experiments performed by Yin and colleagues revealed that when the brains of mice were exposed to 0.1 Gy gamma radiation, it induced alterations in the gene expression involved in neuroprotective functions - most notably DNA repair, cell-cycle control, lipid metabolism, and stress response. Interestingly, later changes also occurred and concerned genes implicated in metabolic function, myelin and protein synthesis, and increases in transcripts for antioxidative enzymes [29,42].

Published studies have also suggested that LDIR may reduce the vulnerability of brain tissues to subsequent exposure to ionizing radiation. Of course, this is largely due to the LDIR-induced adaptive responses, eminently that of antioxidant and anti-inflammatory defense mechanisms. And as indicated by Betlazar et al., these responses are likely reflective of non-linear differential microglial activation, which manifests as an anti-inflammatory or proinflammatory functional state [30].

LDIR Does Not Induce AD

There is still an ongoing debate as to whether LDIR is harmful to the CNS. Many researchers have proposed that ionizing radiation, including both high-dose and LDIR, may trigger mechanisms that ultimately favor the development of AD [43]. Considering the increasing clinical use of diagnostic instruments that emit ionizing radiation, these concerns do remain valid. However, on the basis of more updated scientific literature, it seems that LDIR, specifically at doses less than 100 mGy, does not increase the risk of developing AD and in actuality, may serve as a therapeutic tool for combating neurodegenerative diseases [29].

Interestingly, it has been reported that early transcriptional responses occur in murine brains when exposed to low-doses of x-rays, specifically at 100 mGy. Their results suggested alterations of molecular networks and pathways associated with advanced aging, cognitive function, and AD [44]. However, further investigation into this matter was needed in both the acute and late transcriptional responses, as well as the pathological and cognitive consequences that occur in murine brains after exposure to LDIR.

Another research group exposed C57BL/6J Jms mice to total body irradiation (TBI) using x-rays, specifically at 100 mGy. Hippocampal tissue was collected from the mice and analyzed the expression of 84 related AD genes. Interestingly, mRNA studies showed significant downregulation of only two of the 84 related AD genes - APBB1 and LRP1 - at four hours after irradiation, and of only one gene, IL-1α, at one year after irradiation. However, these studies did not show induction of amyloid fibrillogenesis nor any changes in APP, Aβ, τ, or phosphorylated- τ expression at four months or two years post-radiation. The results showed that TBI induced early and late transcriptional alterations in only a few AD-related genes but did not significantly affect spatial learning, memory, or AD-like pathological changes in mice [40]. Based upon these findings, it was suggested that radiation-induced changes in the expression of genes associated with AD are not necessarily predictors of the emergence of AD.

LDIR as a potential therapeutic modality in AD

In recent years, multiple scientific journals were published assessing the efficacy of ionizing radiation in AD. Prior research has investigated the effects of cranial x-ray radiation, at higher doses, in a transgenic mouse model for AD. The results showed that mice exposed to single x-ray doses reduced the number and size of Aβ plaques. Upon fractionating the doses, specifically at 2 Gy x 5, the researchers noted significant reductions in AD-associated Aβ plaques with subsequent improvement in their cognitive function [45]. Indeed, it was the results of this research that prompted other investigators to explore the possible effects of LDIR on Alzheimer's.

A recent study reported the beneficial effects of LDIR in human Aβ42 expressing drosophila melanogaster AD models. In fact, ionizing radiation at dosages of 50 mGy suppressed AD-like phenotypes, including developmental defects and locomotive dysfunction. Additionally, using the same dose of γ-irradiation reduced Aβ42-induced cell death through downregulation of the Wrinkled gene, which encodes a protein that activates caspases. Ultimately, the authors suggested that LDIR may have hormetic effects on the pathogenesis of Aβ42-associated AD [46].

More importantly, innovative work in the field of low-dose radiation as a therapeutic intervention for AD was performed in 2015. A human subject, who was at the time 81 years of age, began exhibiting symptoms of dementia roughly 10 years prior to being evaluated. The disease progressed gradually to the final stages of AD, and the patient was placed in hospice care in April 2015. A neuropsychological exam was performed one month later, which revealed that the patient was completely unresponsive, only uttering single words, resulting in the patient's spouse seeking therapeutic care [47]. 

The patient was approved for clinical trials and received five computed tomography (CT) scans to the brain over a period of three months, with each scan emitting approximately 40 mGy of ionizing radiation. The first two scans were performed in July 2015. Remarkably, two days after receiving both scans, the patient's condition significantly improved. The patient attempted to speak and expressed her desire to ambulate from her wheelchair. The patient received her third and fourth CT scans in August 2015. The patient's condition continued to improve after the administration of both scans, and it was deemed that the patient had partially restored her memory, speech, movement, and cognition. In November 2015, the patient was deemed no longer eligible for hospice care due to improvements in both her cognition and physical condition. Ultimately, it was suggested that the current mechanism behind LDIR therapeutic effects against AD is a radiation-induced upregulation of adaptive protective systems [47].

Interestingly, the investigators of this study deemed that the cumulative radiation dose of the first four CT scans, which in actuality totaled to 168 mGy, was in the range for radiation-induced optimal health effects. However, upon having the patient exposed to the fifth CT scan, the cumulative radiation dose increased beyond optimal levels, such that it resulted in the patient's cognitive and physical decline. Of course, the patient continued to slowly recover from her setback, and in January 2016, the patient was evaluated once more with noted cognitive and physical improvement [47]. Thus, it is worth suggesting that there may be a biphasic dose-response curve for using brain CT scans in AD.

In a follow-up article released by the same group, the same patient's family requested for the patient to receive ongoing "booster" CT scans every four to five months due to her physical and cognitive improvements. From February 2016 to December 2016, the patient received an additional four CT scans to the brain, with each scan emitting exactly 40 mGy of ionizing radiation [48]. Upon receiving booster CT scans during 2016, it was documented that her condition was slowly but continuously improving. The patient was capable of feeding herself and was able to express emotions, as well as mobility improvements. The patient received an additional two CT scans in January 2017 and sadly began to decline, resulting in a return to hospice care. This finding, which was very similar to the previous year, may provide further evidence for a biphasic response. 

Over a year past, a single CT scan (40 mGy) was administered in July 2017. After receiving her July interval scan, no immediate physical improvement was visualized. However, in October 2017, she showed positive progress once again. In fact, the patient was able to chew and swallow her meals. She displayed facial expressions and appeared relatively happy. Throughout the remainder of 2017, the patient continued to show improvements and was often noted to be smiling and laughing, and continued to demonstrate no difficulty with swallowing [49]. Despite there being setbacks with regards to the patient receiving LDIR for the treatment of severe AD, the results were overall promising. In fact, so much so that this prompted the group to conduct a pilot clinical trial to examine the effects of LDIR in patients with severe AD.

The pilot clinical trial was registered and approved in July 2018 and consisted of four participants who were residents of long-term care facilities. The participants were noted to have been clinically stable for at least three months prior to being selected. The four participants each received three standard CT brain scans, with the first scan emitting a dose of 80 mGy and the following two scans emitting a dose of 40 mGy. [50]. Each scan was scheduled two weeks apart. Qualitative and quantitively changes in cognition, communication, and behavior were subsequently observed, which revealed improvements in three of the four patients. It should be noted that patients showed greater improvement in cognition and behavior after being exposed to the CT scans emitting 80 mGy. In fact, the improvements were noticeable within hours and peaked within five days of receiving treatment. Of course, this pilot study had several limitations. For example, the sample sizes were relatively small, and there were no placebo groups. Additionally, only two different dosages of radiation were utilized in this study. It may be more fruitful for future studies to investigate the therapeutic effects with a greater variety of doses. Nonetheless, this pilot study was considered positive and improved the conditions of patients with severe AD. In fact, the results from this clinical trial suggest that LDIR may, in fact, be a promising therapy for the treatment of neurodegenerative diseases and especially that of Alzheimer's dementia [50].

## Conclusions

Despite significant advancements in the understanding of AD pathology, Alzheimer’s disease is a complex, debilitating neurodegenerative disease that still remains incurable. Current therapeutic modalities are designed to alleviate symptoms and possibly delay the advancement of the disease. Unfortunately, the likelihood of discovering one drug or intervention to successfully treat AD is highly improbable, and therefore, AD will continue to pose as a major public health concern for Americans.

In recent times, low-dose ionizing radiation (LDIR) has gained significant traction from both clinicians and researchers as a valuable therapeutic tool for the treatment of neurodegenerative diseases. Of course, the question as to whether LDIR exposure is harmful to the CNS is still valid to this day.

We discuss the potential for LDIR to induce repair mechanisms against CNS pathology, reduce oxidative stress, stimulate defenses against neuroinflammation, and may have hormetic effects on AD pathogenesis. With no curative medications available in the market, LDIR can provide a source of hope. Albeit controversial, LDIR has shown great promise in treating severe AD patients and we recommend for research groups to investigate both mild and severe cases of AD along with testing biological markers associated with the disease.
